# Infectivity of Scarlet Fever

**Published:** 1882-06

**Authors:** 


### INFECTIVITY OF SCARLET FEVER.

At a meeting of the "Society of Medical Officers of Health" (*British Medical Journal*) Dr. Alfred Carpenter read a paper on some of the causes which increased or modified the infectivity of scarlatina. He considered that a long period of quarantine—such as eight or ten weeks, and sometimes four months—was to be deprecated in the case of children recovering from scarlatina ; he himself, for many years, had isolated cases for a fortnight, and in some instances for a week only, after the departure of the fever, and he never heard of any evil results accruing from such a course. He next mentioned various cases in large schools, which served to explain his views as to the causes of scarlatina. The first outbreak of which Dr. Carpenter made mention was, in his opinion, entirely caused by a cesspool situated in the school yard, which was the recipient of washings from a slaughter-house ; and, once a week at least, the aërial contents of this cesspool were displaced into the closets of the boys, situated in close proximity to it. As soon as this cesspool was emptied, and the drains properly directed into the sewer, scarlatina ceased to appear in the school. Want of proper ventilation in the soil pipe and drains belonging to the building caused an outbreak of scarlatina in another school—the drains being in direct communication with the main sewer of the district, and scarlatina being also at that time very prevalent in the neighborhood. Dr. Carpenter believed that the nature of the blood into which the poison was received aided more materially in increasing the mortality from scarlatina than the character of the poison itself ; and in support of this theory, he instanced two outbreaks of this disease, in one of which the mortality had been excessive, fourteen persons having died out of seventy-five who were attacked ; and in the other outbreak only three deaths occurred, though the total number of cases was one hundred and eight. Dr. Carpenter attributed this difference in the mortality to the surroundings of the children ; in the one instance, the conditions were, on the whole, healthy ; while, in the other, the children were exposed for a much longer period to an impure atmosphere, and were badly cared for at home. The cause of the two outbreaks was similar in character ; the excreta of the first case of scarlatina, in each school, passed down unventilated drains, which communicated directly either with class-rooms inhabited by the children, or with closets used by them. In conclusion, Dr. Carpenter said that he believed scarlatina more often arose from sewage emanations, or from sewage contaminated with scarlatinal germs, than from personal contact. He considered that, in many cases where the cause of the disease had not been discovered, it might have been found, on careful investigation, to proceed from some accidental inhalation of sewer-air ; and that persons who were constantly exposed to such an atmosphere were liable to more severe attacks of scarlatina and diphtheria than those who inhaled the poison for a short period only. Dr. Carpenter also suggested that the discharge of hot water and waste from steam engines into the sewers was a source of danger, in producing scarlatina and diphtheria.—*Medical and Surgical Report.*

